# Machine Learning Explains Long-Term Trend and Health Risk of Air Pollution during 2015–2022 in a Coastal City in Eastern China

**DOI:** 10.3390/toxics11060481

**Published:** 2023-05-25

**Authors:** Zihe Qian, Qingxiao Meng, Kehong Chen, Zihang Zhang, Hongwei Liang, Han Yang, Xiaolei Huang, Weibin Zhong, Yichen Zhang, Ziqian Wei, Binqian Zhang, Kexin Zhang, Meijuan Chen, Yunjiang Zhang, Xinlei Ge

**Affiliations:** 1 Collaborative Innovation Center of Atmospheric Environment and Equipment Technology, Jiangsu Key Laboratory of Atmospheric Environment Monitoring and Pollution Control, School of Environmental Science and Engineering, Nanjing University of Information Science and Technology, Nanjing 210044, China; qzh457814650@163.com (Z.Q.); 18603637718@163.com (Z.Z.); zyc17720568392@163.com (Y.Z.); bqzhang18@163.com (B.Z.);; 2 Lianyungang Environmental Monitoring Center, Lianyungang 222000, Chinalygzwb@163.com (W.Z.)

**Keywords:** air quality, emission, meteorological impact, machine learning, coastal city

## Abstract

Exposure to air pollution is one of the greatest environmental risks for human health. Air pollution level is significantly driven by anthropogenic emissions and meteorological conditions. To protect people from air pollutants, China has implemented clean air actions to reduce anthropogenic emissions, which has led to rapid improvement in air quality over China. Here, we evaluated the impact of anthropogenic emissions and meteorological conditions on trends in air pollutants in a coastal city (Lianyungang) in eastern China from 2015 to 2022 based on a random forest model. The annual mean concentration of observed air pollutants, including fine particles, inhalable particles, sulfur dioxide, nitrogen dioxide, and carbon monoxide, presented significant decreasing trends during 2015–2022, with dominant contributions (55–75%) by anthropogenic emission reduction. An increasing trend in ozone was observed with an important contribution (28%) by anthropogenic emissions. The impact of meteorological conditions on air pollution showed significant seasonality. For instance, the negative impact on aerosol pollution occurred during cold months, while the positive impact was in warm months. Health-risk-based air quality decreased by approximately 40% in 8 years, for which anthropogenic emission made a major contribution (93%).

## 1. Introduction

Air pollution is one of the most important global environmental problems, which can significantly affect human health [1]. Air pollutants can be generally classified into two phases, i.e., gases including ozone (O_3_), nitrogen oxide (NOx), carbon monoxide (CO), sulfur dioxide (SO_2_), and volatile organic compounds (VOCs), and particles, such as fine particulate matter (PM_2.5_) and inhalable particulate matter (PM_10_) [2,3]. Exposure to ambient particulate matter is one of the most important health risk factors [4]. It was estimated that approximately 4.2 million deaths were attributed to particulate matter air pollution all over the world in 2015 [1].These air pollutants can be generated from natural [5] and/or anthropogenic [6,7] sources [2]. Concentrations of air pollutants in ambient air are affected by two major factors, including emissions and meteorological conditions [8,9,10,11]. It is essential to evaluate the impact of these two factors on the variability of air pollution in the atmosphere for making emission control policies.

Relatively serious air pollution is mainly distributed in developing and populous countries, such as China. Air pollution is generally characterized by complicated mixtures along with complex chemical reactions [12,13,14,15] and pollution–weather interactions [10,16,17]. To improve air quality, the Chinese government has been implementing a series of clean air actions since 2013 [18]. Emission inventory studies showed that most pollutant emissions have significantly decreased due to strict emission reductions [19], except for emissions in VOCs and ammonia (NH_3_), which showed a stable but even a slight increase from 2013 to 2017 [20]. Among the typical air pollutants, SO_2_ showed the most significant decrease over China due to efficient emission reduction controls for the power plant sector [20]. Due to emission reductions, substantial improvement in air quality in China has been widely observed, which could prove the effectiveness of the regional emission reduction [20] in controlling air pollution [18,21,22,23].

Recently, many studies applied an air quality model to assess the contribution of anthropogenic emissions and meteorology to changes in air quality during the recent years in China [18,24,25,26]. They found that anthropogenic emission reduction was a dominant driver of air quality improvement. For example, Zhang et al., (2019) found that population-weighted annual mean PM_2.5_ concentrations decreased from 61.8 to 42.0 μg m^−3^ from 2013 to 2017 over China, with dominant contributions (approximately 91%) from anthropogenic emission abatements. Furthermore, they also found that the improvement in PM_2.5_ air pollution was driven by three measures, including strengthening industrial emission standards, upgrades on industrial boilers, phasing out outdated industrial capacities, and promoting clean fuels in the residential sector [18]. However, an increase in summertime ozone was also widely observed during 2013–2017, especially in the North China Plain, which was demonstrated by changes in anthropogenic emissions [27] and a rapid decline in ambient PM_2.5_ concentrations that could slow down the aerosol sink of hydroperoxyl (HO_2_) radicals [28,29]. Those previous studies using chemical transport models have improved our understanding of the response of air quality to changes in emissions and meteorological conditions. However, there is a challenge for a chemical transport model method for evaluating an up-to-date time period due to lack of up-to-date emission inventory data. Recently, some studies tried to use statistical model methods, such as multiple linear regression [28,30,31,32], Kolmogorov–Zurbenko filters [33,34], and random forest [11,35,36,37,38,39,40], to separate the contribution of anthropogenic emission and meteorology to trends in air quality. Two of the advantages of statistical models are there is no need for emission inventory data as model input and there is no need for complex chemical and physical mechanisms in the model. Generally, a statistical model could be built by using observed air quality and meteorological data, which, therefore, could offer up-to-date outputs to meet the observation timing. Among these statistical model methods, the random forest (RF) algorithm—which is one of the machine learning algorithms—exhibited a robust performance with high noise immunity and high accuracy, which is also able to handle high-dimensional data and nonlinear problems [35,41,42].

In addition to emissions, meteorological conditions also play an important role in affecting air quality, especially in the typical coastal regions. However, the impact of meteorological conditions on air quality at a typical coastal region was not well quantified. In this study, we applied a machine learning approach to evaluate the impact of anthropogenic emissions and meteorology on trends in air quality during 2015–2022 in a coastal city of eastern China (i.e., Lianyungang). Additionally, drivers of health-risk air quality index (HAQI) and the corresponding premature mortality from 2015 to 2022 were also investigated.

## 2. Data and Methods

### 2.1. Data Source

**Figure 1:**
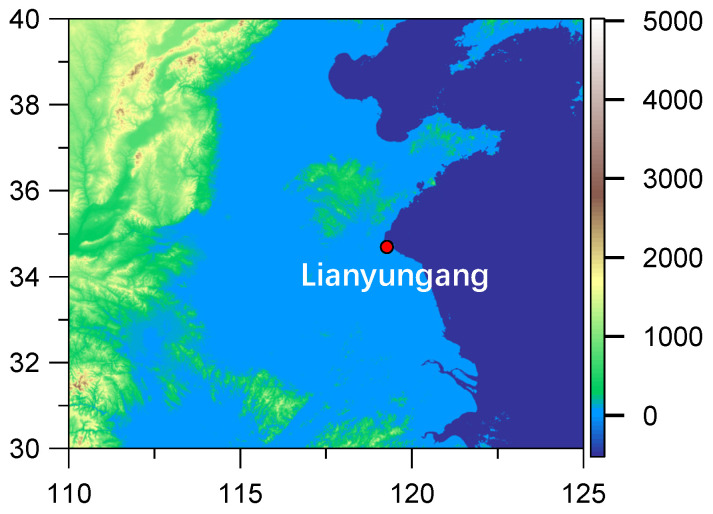
Geographic location (red dot) of the coastal city (Lianyungang) in eastern China.

In the present study, the hourly mass concentration of air pollutants, including PM_2.5_, PM_10_, CO, NO_2_, O_3_, and SO_2_, in Lianyungang, which is a typical coastal city in eastern China, was obtained from the China National Environmental Monitoring Center network (https://quotsoft.net/air, last access 10 March 2023). The meteorological parameters were taken from ERA5 reanalysis data of the European Centre for Medium-Range Numerical Prediction (ECMWF), which is accessible at https://cds.climate.copernicus.eu/ (last access 10 January 2023). The meteorological data include zonal wind speed (U10), meridional wind speed (V10), temperature (T), boundary layer height (BLH), solar radiation (SR), sea level pressure (SP), total cloud coverage (TCC), total precipitation (TP), relative humidity (RH), zonal wind speed of 500 hpa (U500), meridional wind speed of 500 hpa (V500), 500 hpa vertical wind speed (W500), 850 hpa zonal wind speed (U_850), 850 hpa meridional wind speed (V850), and 850 hpa vertical wind speed (W850). The horizontal and temporal resolutions of these meteorological data were 0.25 degree and 1 h, respectively. The geographic location of Lianyungang is shown in Figure 1.

### 2.2. Meteorological Normalization Using RF Model

In the present study, a meteorological normalization approach based on the RF algorithm [35] was applied to separate the impact of anthropogenic emissions and meteorological conditions on trends in air pollutants, including PM_2.5_, PM_10_, CO, NO_2_, O_3_, and SO_2_, respectively. A more detailed description of such meteorological normalization approach can be found elsewhere [35,36,37,42]. The daily average data were used for the RF model analysis, which was consistent with Grange et al., (2018) [35]. Briefly, the prediction features for the RF model included time variables (i.e., proxy of emissions) and meteorological parameters. To explain long-term, seasonal, and weekly variations in anthropogenic emissions, three corresponding time variables were used here, which were Unix time, Julian day (day of year), and weekdays (day of week), respectively. The RF model prediction features for the meteorological field include U10, V10, T, BLH, SR, TCC, TP, RH, U500, V500, W500, U850, V850, and W850, which could explain the impact of these meteorological parameters on variations in air pollution. The day of the week variable was a categorical variable, while all the other variables were numeric variables. In practice, a specific RF model for an individual air pollutant (e.g., PM_2.5_, PM_10_, CO, NO_2_, O_3_, or SO_2_) was first established. The 70% and 30% of the input data were divided as training and testing data sets, respectively. In this processing, the RF model’s built-in importance indexes could be obtained. In addition, a fivefold cross validation was applied to evaluate the model performance. The normalized mean square error (NMSE), root mean square error (RMSE), and correlation coefficient between observation and prediction were calculated for evaluation metrics.

To obtain weather-normalized (so-called deweathered) prediction, the total 1000-time prediction was achieved. To do so, the model input data for daily meteorological variable features were randomly resampled from the historical weather data during 2001–2022, while time variables were resampled. This process was repeated in the corresponding 1000 predictions. For each processing, a specific model prediction result was then taken. The final deweathered concentration for the specific air pollutant was obtained by aggregating the 1000 predictions using arithmetic mean.

In addition, we applied a meteorological impact index method to evaluate the extent of meteorological influence on the concentrations of air pollution in different years and/or months. This method has been described in a previous study [34]. Briefly, the relative difference in the monthly mean concentration of an individually observed and deweathered air pollutant was due to the meteorological impact. The meteorological (MET) impact index could be calculated using Equation (1). In Equation (1), $C_{i,observed}$ and $C_{i,deweathered}$ represent the monthly mean concentrations of an observed and deweathered air pollutant, respectively, where *i* refers to individual month. The model configuration used in this study is consistent with previous studies [35]. In this work, the RF modeling analysis was performed using a random forest R package based on the R language [43].

(1) $$\mathrm{MET}\;\mathrm{impact}\;\mathrm{index}=\frac{C_{i,observed}-C_{i,deweathered}}{C_{i,observed}}$$

### 2.3. Calculation of Health-Risk-Based AQI (HAQI)

In this paper, we also calculated the HAQI value to evaluate the health impact of air pollution. A detailed calculation method can be found in Hu et al., (2015) [44]. Meanwhile, we also evaluated the impact of anthropogenic emissions and meteorological conditions on changes in HAQI from 2015 to 2022. More discussion will be given in the next section. Relative risk (RR) was used to estimate the health effect for the six air pollutants (including PM_2.5_, PM_10_, NO_2_, SO_2_, CO, and O_3_) by using Equation (2). The RR based on the equivalent concentration of the pollutants $\left({\mathrm{RR}}^{'}\right)$ was calculated by Equation (3):

(2) $$\mathrm{RR}=\exp\left\{\beta \left(C-C_{0}\right)\right\},\;C>C_{0}$$

where β refers to the exposure–response relationship coefficient, C represents the concentration of each pollutant, and C_0_ is the threshold concentration, below which the pollutant proves no obvious adverse health effects:

(3) $${\mathrm{RR}}^{'}={\mathrm{ER}}_{\mathrm{Total}}+1$$

where ${\mathrm{ER}}_{\mathrm{Total}}$ refers to the sum of the total excess risk for simultaneous exposure to the six air pollutants. Equation (4) introduces the equivalent pollutant concentration of the *i*th criteria pollutant ($C_{i}\prime$):

(4) $${C_{i}}^{\prime }=\frac{\mathrm{In}\left({\mathrm{RR}}^{\prime }\right)}{\beta_{i}}+C_{0,i}$$

where $\beta_{i}$ represents the exposure–response relationship coefficient of the ith pollutant, and $C_{0,i}$ is the threshold concentration of the *i*th pollutant. Then $C_{i}^{\prime }$ can be used to calculate the equivalent concentration of the ith criteria pollutant (${\mathrm{HAQI}}_{i}$), which is shown in Equations (5) and (6):

(5) $${\mathrm{HAQI}}_{i}=\frac{\left({\mathrm{AQI}}_{i,j}-{\mathrm{AQI}}_{i,j-1}\right)}{\left(C_{i,j}-C_{i,j-1}\right)}\times \left(C_{i}\prime -C_{i,j-1}\right)+{\mathrm{AQI}}_{i,j-1},\;j>1$$

(6) $${\mathrm{HAQI}}_{i}={\mathrm{AQI}}_{i,1}\frac{C_{i}\prime }{C_{i,1}},\;j=1$$

where j is the health category index; $C_{i,j}$ and $C_{i,j-1}$ represent the upper-limit concentrations for the jth and j-1th health categories; and ${\mathrm{AQI}}_{i,j}$ and ${\mathrm{AQI}}_{i,j-1}$ refer to the air quality index of the pollutant that corresponds to $C_{i,j}$ and $C_{i,j-1}$.

Finally, Equation (7) shows that the overall HAQI is determined by the maximum of all ${\mathrm{HAQI}}_{i}$s.

(7) $${\mathrm{HAQI}}_{i}=\max\left({\mathrm{HAQI}}_{1},{\mathrm{HAQI}}_{2},\ldots ,{\mathrm{HAQI}}_{n}\right),\;n=1,\;2,\;\ldots ,\;6.$$

### 2.4. Calculation of Premature Mortality (M)

The premature mortality (M) attributable to PM_2.5_ and O_3_ can reflect health effects of atmospheric pollutants. A detailed calculation method can be found in Apte et al., (2015) [45]. Briefly, the premature mortality is calculated in Equation (8):

(8) $$M=Y_{o}\times \mathrm{Pop}\times \frac{\mathrm{RR}-1}{\mathrm{RR}}$$

where $Y_{o}$ refers to the baseline mortality rate corresponding to a particular disease category in regions, which can be found in the Statistical Yearbook of Public Health and Family Planning in China. Pop represents the population for this region in 2015. RR has been calculated in Equation (2), and $\frac{\mathrm{RR}-1}{\mathrm{RR}}$ here refers to the attribution fraction, which is attributed to respiratory disease linked to O_3_ and stroke, ischemic heart disease (IHD), chronic obstructive pulmonary disease (COPD), and lung cancer (LC) linked to PM_2.5_.

## 3. Results and Discussion

### 3.1. Modeling Evaluation

**Figure 2:**
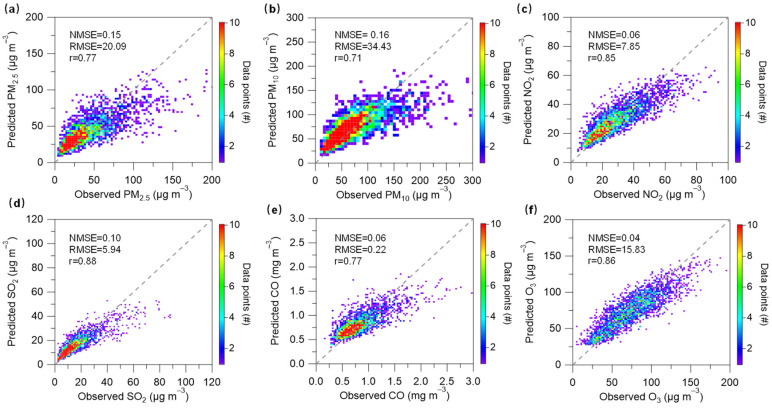
Fivefold cross validation between the RF-predicted concentration with the observed concentration for (**a**) PM_2.5_, (**b**) PM_10_, (**c**) NO_2_, (**d**) SO_2_, (**e**) CO, and (**f**) O_3_, respectively.

Figure 2 presents the fivefold cross-validation results of the RF models for the six air pollutants (including PM_2.5_, PM_10_, NO_2_, SO_2_, CO, and O_3_). Overall, the predicted concentrations of all air pollutants were correlated well (*r* = 0.71–0.88) within the observed concentrations. The NMSE values of the six air pollutants were in the range of 0.04–0.16. The corresponding RMSE values were in the range of 0.22–34.43. These model validation results could prove the good performance for the RF models.

**Figure 3:**
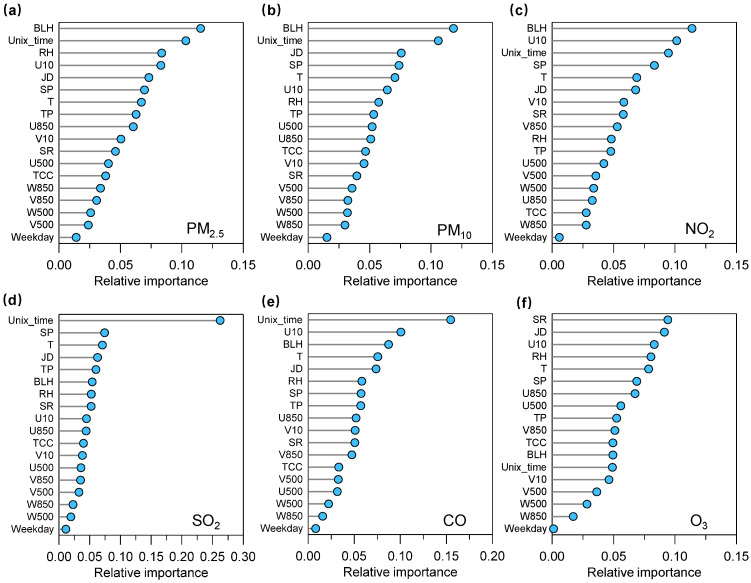
Relative importance (mean decrease impurity) of the prediction features resolved by the RF models for (**a**) PM_2.5_, (**b**) PM_10_, (**c**) NO_2_, (**d**) SO_2_, (**e**) CO, and (**f**) O_3_.

Figure 3 shows the relative importance taken from the RF model for an individual air pollutant. For most air pollutants (i.e., PM_2.5_, PM_10_, NO_2_, SO_2_, and CO), the Unix time—as a long-term anthropogenic emission proxy—shows highly relative importance. This reveals the important impact of anthropogenic emission on trends in air pollutants (i.e., PM_2.5_, PM_10_, NO_2_, SO_2_, and CO) during 2015–2022. However, the Unix time shows a relatively low contribution to the relative importance, suggesting that the variability of atmospheric ozone in Lianyungang was not mainly driven by anthropogenic emissions.

### 3.2. Impact of Anthropogenic Emissions on Air Pollution Trends

**Figure 4:**
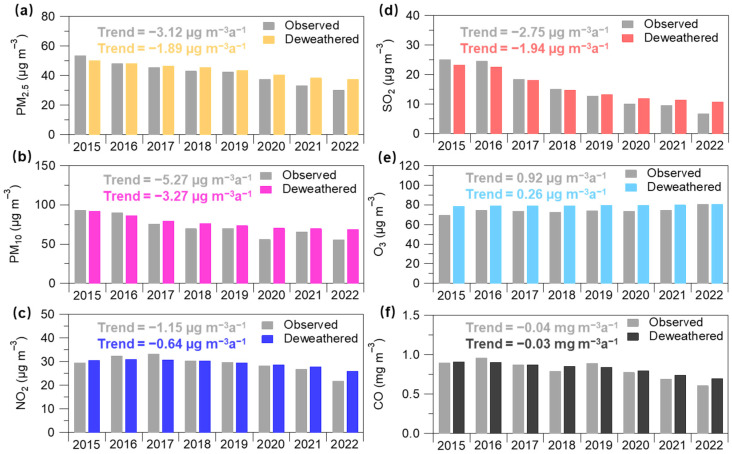
Annual mean concentrations of observed and deweathered (**a**) PM_2.5_, (**b**) PM_10_, (**c**) NO_2_, (**d**) SO_2_, (**e**) O_3_, and (**f**) CO from 2015 to 2022, respectively.

Figure 4 presents the annual mean concentrations of the deweathered and observed six air pollutants during 2015–2022. The observed SO_2_ concentration showed the largest difference in its concentrations from 2015 to 2022 among the six air pollutants, which decreased from 25.24 to 6.87 µg m^−3^. The observed PM_2.5_ and PM_10_ concentrations decreased from 53.70 and 93.31 to 30.52 and 55.84 µg m^−3^ in 8 years, respectively. The observed NO_2_ showed a slight decrease during 2015–2022. The observed SO_2_ showed the largest decreasing trends (−2.75 µg m^−3^ a^−1^), followed by PM_2.5_ (−3.12 µg m^−3^ a^−1^), PM_10_ (−5.27 µg m^−3^ a^−1^), CO (−0.04 mg m^−3^ a^−1^), and NO_2_ (−1.15 µg m^−3^ a^−1^). The corresponding deweathered concentrations for these air pollutants showed similar trends, i.e., SO_2_ (−1.94 µg m^−3^ a^−1^), PM_2.5_ (−1.89 µg m^−3^ a^−1^), PM_10_ (−3.27 µg m^−3^ a^−1^), CO (−0.03 mg m^−3^ a^−1^), and NO_2_ (−0.64 µg m^−3^ a^−1^). These results reflect a large contribution (55–75%) of anthropogenic emissions to the changes in air quality trend during 2015–2022. However, the deweathered O_3_ presented an increasing trend during 2015–2022, highlighting the continuous aggravation of O_3_ air pollution in this region. This is overall consistent with some previous observation and modeling studies [28,46,47].

**Figure 5:**
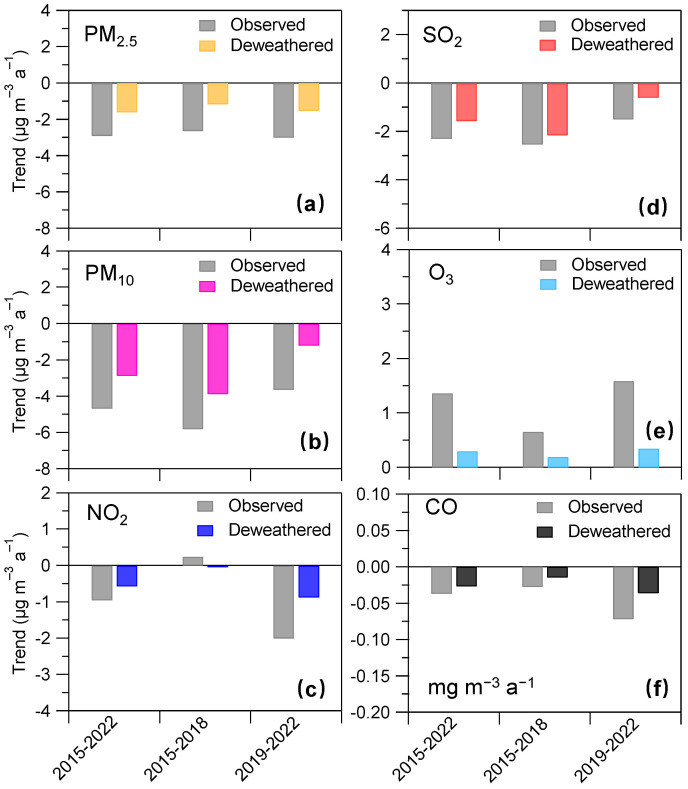
Trends in (**a**) PM_2.5_, (**b**) PM_10_, (**c**) NO_2_, (**d**) SO_2_, (**e**) O_3_, and (**f**) CO during the three periods, i.e., 2015–2022, 2015–2018, and 2019–2022, respectively.

Since 2013, the Chinese government has implemented a series of policies to improve air quality, such as the Action Plan for Air Pollution Prevention and Control in 2013 and the 3-year Action Plan for Winning the Battle against Blue Skies in 2018. To explore the difference in trends of air quality during the different time periods associated with the different air pollution control measures [18], we compared the trends in the observed and deweathered concentrations of the six air pollutants during 2015–2022, 2015–2018, and 2019–2022, respectively (see Figure 5). Overall, the six air pollutants presented different trends at different time periods. For instance, trends in deweathered PM_2.5_ concentrations were −1.89 µg m^−3^ a^−1^, −1.54 µg m^−3^ a^−1^, and −2.15 µg m^−3^ a^−1^ during 2015–2022, 2015–2018, and 2019–2022, respectively. The deweathered NO_2_ trend showed a similar trend with a more rapid reduction during the latter period (−1.04 µg m^−3^ a^−1^) rather than the former period (−0.06 µg m^−3^ a^−1^). These results could be attributed to more rapid reductions in PM_2.5_ and NO_2_ from 2020 to 2022. The deweathered SO_2_ showed a larger reduction during 2015–2018 than that during 2019–2022, highlighting a more effective reduction in SO_2_ during the former period. This is also consistent with emission inventory studies [18], where they also found substantial emission reduction of SO_2_ over eastern China. The deweathered PM_10_ also presented a larger annual reduction ratio (−5.35 µg m^−3^ a^−1^) during 2015–2018 than that during 2019–2022, which could be partly explained by mankind dust emission control (such as urban road dust emissions) [48,49]. During these three periods, the deweathered O_3_ presented a comparable trend with a range of 0.21–0.37 µg m^−3^ a^−1^, which reflects the continuity of the O_3_ pollution trend during the different periods in this region. It should be noted that an unexpected short-term emission reduction due to the COVID-19 pandemic lockdown in the spring of 2020 in eastern China has been widely reported [38,50,51,52,53,54], which might have influence on the long-term trend observed in the present study. Due to the methodology limitation, we here roughly evaluate such influence. To do so, the data in 2020 were replaced by that in 2019. The comparison between the original trends and the modified trends by using the replaced data could represent the upper limit of the COVID-19 impact. The modified trends in observed and deweathered concentrations of air pollutants during 2015–2022 were −3.00 and −1.87 µg m^−3^ a^−1^, −5.03 and −3.24 µg m^−3^ a^−1^, −1.07 and −0.63 µg m^−3^ a^−1^, −2.71 and −1.93 µg m^−3^ a^−1^, 0.91 and −0.26 µg m^−3^ a^−1^, and −0.04 and −0.03 mg m^−3^ a^−1^ for PM_2.5_, PM_10_, NO_2_, SO_2_, O_3_, and CO, respectively. The difference between the original trends (see Figure 4) and the modified trends in observed and deweathered concentrations was very small or even negligible (1–7% and 0–2%, respectively). These results suggest that the short-term emission reduction during the COVID-19 pandemic lockdown could influence the long-term trend during 2015–2022, but the magnitude of the influence might be small. Moreover, it would be more helpful to evaluate such influence by using chemical transport modeling, which could be further performed in the future.

### 3.3. Impact of Meteorology on Air Pollution Trends

**Figure 6:**
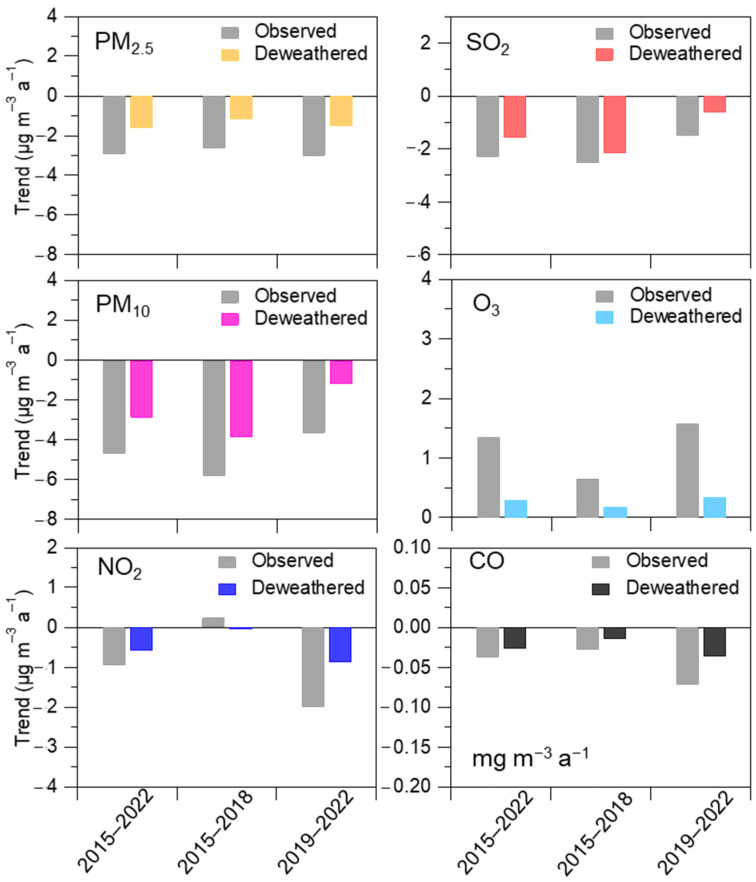
Variability of meteorological (MET) impact index for (**a**) PM_2.5_, (**b**) PM_10_, (**c**) NO_2_, (**d**) SO_2_, (**e**) O_3_, and (**f**) CO during the three periods, i.e., 2015–2022, 2015–2018, and 2019–2022, respectively. Error bars refer to the standard deviation of the yearly mean MET impact index values during each period.

**Figure 7:**
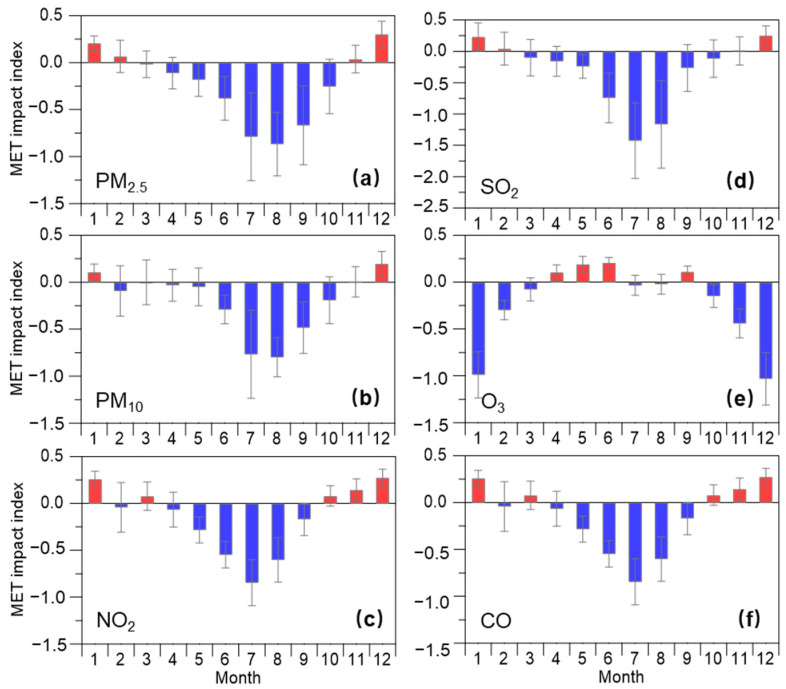
Monthly variability of meteorological (MET) impact index for (**a**) PM_2.5_, (**b**) PM_10_, (**c**) NO_2_, (**d**) SO_2_, (**e**) O_3_, and (**f**) CO, respectively. Error bars refer to the standard deviation of the monthly mean MET impact index values during each period.

To understand the impact of meteorological conditions on trends in air quality, we further quantified an annual and seasonal mean meteorological impact index, a proxy for the potential contribution of the meteorological impact to changes in concentrations of air pollutants (see Figure 6 and Figure 7). The positive value for the meteorological impact index could reflect negative meteorology, that is, adverse meteorological conditions for air pollution, while the negative values for that reflect those favorable meteorological conditions for improving air quality [36,37,42]. As shown in Figure 6, the meteorological impact index presented negative values for all six air pollutants during different time periods, suggesting favorable meteorological conditions that play an important positive impact on air quality in this coastal city region. Except for O_3_, the magnitudes of such values for most air pollutants were larger during 2019–2022 than that during 2015–2018, suggesting that meteorological conditions (such as atmospheric diffusion conditions and/or air temperature) during the latter period were more favorable for reducing concentrations of air pollutant than during the former period. Except for O_3_, the meteorological impact index of the five air pollutants presents relatively high values during warm months, while it presents relatively low values and even positive values during cold months in this coastal city region. These results could provide direct evidence to prove that meteorological conditions during cold months could promote aerosol (i.e., PM_2.5_ and PM_10_) and gas (i.e., SO_2_, NO_2_, and CO) pollution. The meteorological conditions during warm months play an important role in reducing the concentrations of these air pollutants. The meteorological impact index of O_3_ shows an overall opposite variation compared with other air pollutants, which present relatively low and high values during cold months and warm months, respectively. This is in line with the fact that the most frequent O_3_ pollution occurs in warm seasons due to unfavorable meteorological conditions, such as strong solar radiation and high air temperature. This is consistent with the RF built-in importance results with the most important prediction feature for solar radiation and U10 (Figure 3).

### 3.4. Health Risk and Premature Mortality Assessment

**Figure 8:**
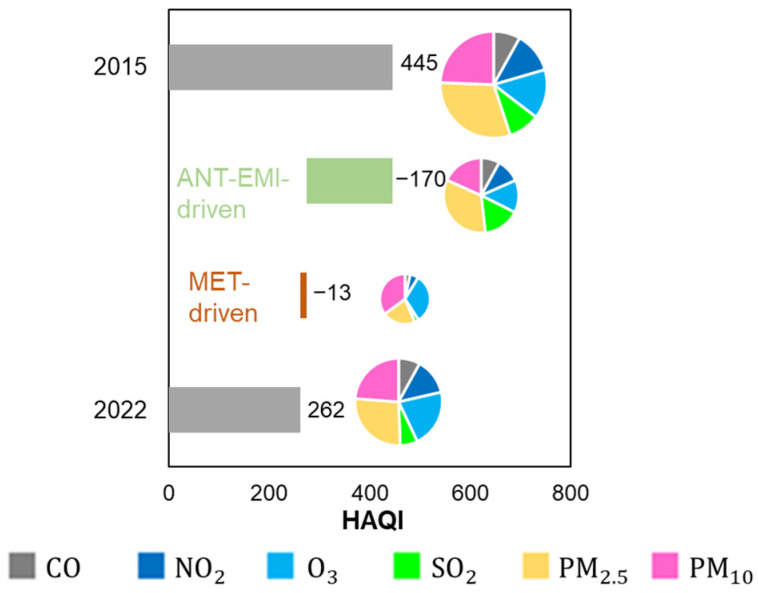
Annually averaged HAQI values in 2015 and 2022 and corresponding drivers including anthropogenic emissions (ANT-EMI-driven) and meteorological variations (MET-driven) for the six criteria pollutants, respectively.

**Figure 9:**
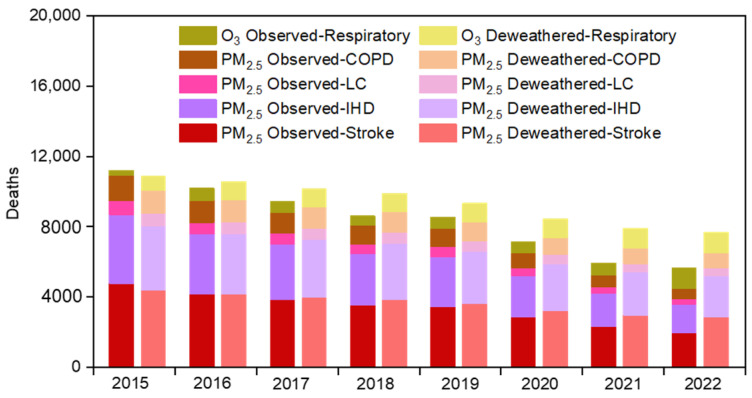
Cause-specific premature mortality from observed and deweathered PM_2.5_ and O_3_ during 2015–2022, respectively. The causes are respiratory disease, COPD, LC, IHD, and stroke.

Figure 8 presents changes in the HAQI values and corresponding drivers (i.e., anthropogenic emissions and meteorology) from 2015 to 2022. The HAQI values decreased from 445 to 262 during 2015–2022. PM_2.5_ and PM_10_ contributed a large fraction (51–55%) to the HAQI in both 2015 and 2022. Anthropogenic emission contributed to approximately 93% of changes in value from 2015 to 2022, where PM_2.5_ and PM_10_ contributed approximately 50% to this emission driver. This suggests that the decline in HAQI value from 2015 to 2022 was driven by anthropogenic emission reduction. As reported by a previous study, approximately 0.894 million premature deaths in 2017 were estimated due to PM_2.5_ and O_3_ pollution across China [55]. To further evaluate health risk associated with long-term exposure to air pollution, we estimated premature mortality from ambient PM_2.5_ and O_3_ pollution in the Lianyungang region. Figure 9 presents trends in cause-specific premature mortality related to observed and deweathered PM_2.5_ and O_3_ from 2015 to 2022, respectively. Among the five specific causes, PM_2.5_-IHD and PM_2.5_-stroke accounted for a major fraction (approximately 71–78%) of the total number of deaths, due to the fact that stroke and IHD dominate the total mortality [45]. The highest premature mortality was attributable to observed PM_2.5_ and O_3_ in 2015, which would cause a total of 11,208 deaths. The specific causes were PM_2.5_-IHD (3931), PM_2.5_-stroke (4764), PM_2.5_-LC (770), PM_2.5_-COPD (1446), and O_3_-respiratory (297). Overall, the observed mortality from PM_2.5_ pollution declined during 2015–2022. The results indicated by the present study were comparable to a previous study, which showed a significant reduction in premature deaths attributable to long-term PM_2.5_ exposures from 2013 to 2016 in China due to emission control [56]. The deweathered PM_2.5_-related mortality was decreased from 10,088 deaths to 6501 deaths in 8 years, while the deweathered O_3_-related mortality was increased from 804 deaths to 1192 deaths. The deweathered PM_2.5_-related mortality presented a decreasing trend (−531 deaths per year). The O_3_-related mortality presented an increasing trend (38 deaths per year). These results suggest that reduction in exposure risk to ambient PM_2.5_ was driven by anthropogenic emission control in this region. This is consistent with the results obtained from chemical transport modeling, which showed that air pollution control avoided 0.39 million deaths caused by PM_2.5_ pollution in China from 2012 to 2017 [57]. However, health risk linked to ambient O_3_ pollution was increased. The difference of the total deweathered deaths from 2015 to 2022 was 3199, suggesting that China’s clean air actions might avoid air-pollution-induced deaths in the Lianyungang region. The relative change in observed and deweathered values reflected the magnitude of the meteorological impact (see Equation (1)). The highest meteorology-driven impact could lead to 2050 deaths, which occurred in 2021. This suggests that changes in meteorological conditions could significantly affect air pollution deaths in this coastal region. Although we have estimated cause-specific premature mortality in this study, a further comparison between estimation and observation in the real world would be more useful to understand the uncertainty of our estimates and more helpful to further understand the health risk of air pollution in a future study.

## 4. Conclusions

In this study, the impact of anthropogenic emissions and meteorology on trends in air quality during 2015–2022 in a coastal city was investigated using an RF modeling approach. The annual mean PM_2.5_, PM_10_, NO_2_, SO_2_, and CO decreased from 53.70 µg m^−3^, 93.31 µg m^−3^, 29.45 µg m^−3^, 25.24 µg m^−3^, and 0.90 mg m^−3^ to 30.52 µg m^−3^, 55.84 µg m^−3^, 21.85 µg m^−3^, 6.87 µg m^−3^, and 0.61 mg m^−3^, during 8 years, respectively. The annual mean concentrations of deweathered PM_2.5_, PM_10_, NO_2_, SO_2_, and CO presented decreasing trends with decreasing rates of −1.89 µg m^−3^ a^−1^, −3.27 µg m^−3^ a^−1^, −1.89 µg m^−3^ a^−1^, −0.64 µg m^−3^ a^−1^, −1.94 µg m^−3^ a^−1^, and −0.03 mg m^−3^ a^−1^, which contributed 61%, 62%, 56%, 71%, and 75% to the observed trends, respectively. The observed and deweathered O_3_ presented an increasing trend with increasing rates of 0.92 µg m^−3^ a^−1^ and 0.26 µg m^−3^ a^−1^, respectively. These results demonstrated the dominant contribution of anthropogenic emission to trends of air pollutants during 2015–2022. The HAQI value decreased from 445 to 262 in 8 years. The contribution of anthropogenic emissions and meteorological conditions to the changes in HAQI were 93% and 7%, respectively. These results suggest that the substantial improvement in overall air quality was driven by anthropogenic emission reduction, which proved the effectiveness of clean air actions on air pollution control in the coastal city. Moreover, the clean air action could avoid 3199 deaths from 2015 to 2022 in the Lianyungang region, highlighting the health benefit of air pollution control policies.

## Data Availability

The data will be made available upon request.
